# Keeping it in the family: Use of the grandmother, mother, and child technique to navigate complex anatomy during Coronary sinus Reducer Implantation

**DOI:** 10.1002/ccd.30858

**Published:** 2023-11-08

**Authors:** Rohin K. Reddy, Michael Foley, Francesco Giannini, Rasha K. Al‐Lamee

**Affiliations:** ^1^ National Heart and Lung Institute Imperial College London London UK; ^2^ Interventional Cardiology Unit IRCCS Ospedale Galeazzi Sant'Ambrogio Milan Italy

**Keywords:** Coronary Sinus Reducer, interventional cardiology, percutaneous intervention

## Abstract

The Coronary Sinus Reducer® (CSR) is an emerging therapy for refractory angina recommended once no further pharmacologic or coronary revascularization options are available. We present the case of a 72‐year‐old man who underwent CSR implantation. Complex coronary sinus anatomy necessitated an innovative “grandmother, mother, and child” catheter approach.

## 1 INTRODUCTION

Clinical guidelines define refractory angina as long‐lasting symptoms (>3 months) secondary to established reversible ischemia in the presence of obstructive coronary artery disease, which remain uncontrolled with maximal medical therapy, bypass grafting, or percutaneous coronary intervention (PCI).^1^ Severe symptoms and limited therapeutic options result in severely impaired physical function and wellbeing.^2^ The Coronary Sinus Reducer® (CSR) is an hourglass‐shaped balloon‐expandable stainless‐steel mesh inserted percutaneously into the coronary sinus and has evidence of efficacy in refractory angina.^3^ Once deployed, the CSR results in permanent focal narrowing of the coronary sinus lumen. We present the case of a patient with refractory angina and complex coronary sinus anatomy that was treated with CSR implantation via utilization of a novel “grandmother, mother and child” technique.

## 2 CASE REPORT

A 72‐year‐old man was referred to cardiology services for treatment of refractory angina by his primary care physician. Relevant cardiac history included coronary artery bypass grafting with left internal mammary artery (LIMA) grafting to the left anterior descending (LAD) artery, and venous grafts to the obtuse marginal artery and first diagonal branch of the LAD. Earlier that year he had a non‐ST elevation myocardial infarction, treated with PCI to the left main stem and circumflex arteries. His past medical history was significant for type 2 diabetes mellitus and peripheral vascular disease.

His quality of life was extremely poor, with severe limitation of daily activities (Canadian Cardiovascular Society [CCS] grade IV) despite up‐titration to maximally tolerated anti‐anginal therapy. Physical examination was unremarkable. Coronary angiography revealed a heavily diseased LAD with calcification in the mid right coronary artery. The LIMA graft and stents remained patent, whilst the venous grafts to the first diagonal and obtuse marginal artery appeared occluded. Cardiac magnetic resonance imaging demonstrated a left ventricular ejection fraction of 48%, with quantitative perfusion mapping demonstrating extensive multivessel perfusion defects sparing only the basal inferoseptum.

Following discussion by a multidisciplinary Heart team there were no conventional revascularization options. He was therefore considered a good candidate for CSR implantation.

A 9 Fr right internal jugular venous sheath was implanted under ultrasound‐guidance. The coronary sinus ostium was engaged using a 6 Fr multipurpose catheter over an 0.035 guide wire, however, this could not be advanced beyond a proximal valve in a highly angulated coronary sinus. A 5Fr AL1 catheter was also able to engage the CS ostium but was also not able to advance. At this stage a “mother and child technique” was utilized. The wire was removed, the hub of the 5Fr AL1 was removed, and a 7Fr AL1 guide catheter was advanced over it to provide more support. With this extra support, the internal 5Fr AL1 catheter was able to advance into the distal coronary sinus and a venogram was acquired.

**Figure 1:**
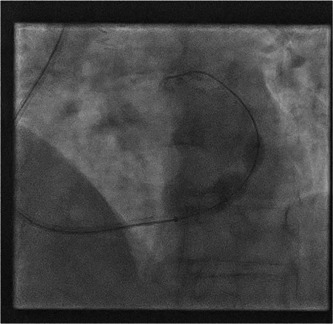
Successful deployment of the Coronary Sinus Reducer®.

The 7 Fr AL1 guide was advanced over the inner 5Fr diagnostic catheter to the midportion of the sinus, which was challenging due to a second valve. We then elected to utilize a grandmother, mother and child technique, using the existing 2 catheters as a supportive rail for delivery of the 9 Fr Cordis guide catheter (Video 1, 1). This was successfully delivered to the mid coronary sinus and the inner 7Fr and 5Fr catheters were removed. The CSR was then advanced (Video 2), positioned and deployed successfully (Figure 1, 1 and Video 3).

**Video 1:** The “grandmother, mother and child” technique, using the 7Fr AL1 inner 5Fr catheters as a supportive rail for delivery of the 9 Fr Cordis guide catheter to advance the system deep within the coronary sinus. Video content can be viewed at https://onlinelibrary.wiley.com/doi/10.1002/ccd.30858

**Video 2:** Advancement of the Coronary Sinus Reducer®. Video content can be viewed at https://onlinelibrary.wiley.com/doi/10.1002/ccd.30858

**Video 3:** Successful deployment of the Coronary Sinus Reducer®. Video content can be viewed at https://onlinelibrary.wiley.com/doi/10.1002/ccd.30858

## 3 DISCUSSION

To our knowledge, this is the first published application of the grandmother, mother, and child technique to CSR implantation. The original mother‐daughter technique was initially developed due to the limitations of poor backup support with a 6 Fr guide catheter compared to 7 or 8 Fr alternatives.^4^ It allows the delivery of stents and balloons past extremely tortuous vessels and calcific lesions where extra support is required. The concept has since been advanced in PCI, with development of the bespoke GuideLiner® system (Vascular Solutions, Inc.), facilitating deep vessel engagement and device delivery, particularly useful in chronic total occlusions. The mother‐and‐daughter technique is associated with good procedural success and rates of stent deformation and coronary dissection have decreased with subsequent iterations.^5^

We are only aware of one prior report describing the use of the grandmother, mother, and child technique in which a 6 Fr GuideLiner® was placed within an 8 Fr Guideliner® to facilitate complex PCI.^6^ With the case presented within this report, we demonstrate that the grandmother, mother, and child technique is both novel and feasible for CSR implantation, particularly useful for the CSR system which must be delivered through a 9 Fr guide catheter.

The operator may wish to take several general steps to minimize complications during CSR implantation with the grandmother, mother, and child technique.^6^ These include avoiding injection through the system to prevent hydraulic dissection and careful catheter back‐bleeding to prevent air entrainment or thrombus embolization. Additionally, operators should be vigilant for signs of hemodynamic instability or ischemia as the triple catheter system may occlude vascular flow.

We present this case and technique because adoption of CSR implantation has risen since its inception and we hope that this report aids operators in future complex cases. The CSR is a rarity for an invasive intervention in medicine, in that randomized double‐blind placebo‐controlled evidence exists supporting its efficacy, demonstrated by improvements in at least two CCS classes compared with sham in the COSIRA trial.^3^ A larger randomized double‐blind placebo‐controlled trial enrolling a more diverse patient population is currently recruiting to replicate these results with a primary efficacy endpoint of exercise duration (NCT05102019). Based on these data, the CSR has been designated a Class IIb recommendation in refractory angina by European Society of Cardiology guidelines and was recently approved for usage in the United Kingdom by the National Institute for Health and Care Excellence.

The putative mechanism of anginal relief is that increased venous drainage pressures produced via focal coronary sinus narrowing results in arterial capillary dilatation, coronary collateral recruitment and redistribution of flow from the less‐ischemic epicardium to the ischemic endocardium.^3^ A paucity of human data underpins this hypothesis. The ORBITA‐COSMIC study (NCT04892537) is an ongoing randomized placebo‐controlled trial that is assessing MRI myocardial perfusion change with the CSR in patients with refractory angina. This trial is seeking to elucidate the mechanism by which the CSR delivers benefit for patients that previously had few therapeutic avenues.

## 4 CONCLUSION

We report the first described case using the grandmother, mother, and child technique to successfully implant a CSR in a technically challenging procedure due to atypical coronary sinus anatomy. As clinical interest in this novel device grows, it is crucial that operators are familiar with accessing the coronary sinus and have techniques in their repertoire to safely manage complex anatomy to ensure optimal outcomes for patients with refractory angina.

## CONFLICT OF INTEREST STATEMENT

The authors declare no conflict of interest.

## Data Availability

Data sharing is not applicable to this article as no new data were created or analyzed in this study.
